# Technical Challenges of Enterprise Imaging: HIMSS-SIIM Collaborative White Paper

**DOI:** 10.1007/s10278-016-9899-4

**Published:** 2016-08-30

**Authors:** David A. Clunie, Don K. Dennison, Dawn Cram, Kenneth R. Persons, Mark D. Bronkalla, Henri “Rik” Primo

**Affiliations:** 1 Pixelmed Publishing LLC., 943 Heiden Rd, Bangor, PA 18013 USA; 2 Don K Dennison Solutions Inc., 205 Fern Cres, Waterloo, ON N2V 2P9 Canada; 3 Department of Information Technology, University of Miami Health System, Miami, FL 33136 USA; 4 Mayo Clinic and Foundation, 200 First St. SW, Pb 2-58, Rochester, MN 55905 USA; 5 Merge Healthcare, 900 Walnut Ridge Drive, Hartland, WI 53029 USA; 6 Digital Health Services, Siemens Healthineers, 65 Valley Stream Parkway, Malvern, PA 19355 USA

**Keywords:** Anatomic pathology, Archive, Dermatology, Digital image management, Digital Imaging and Communications in Medicine (DICOM), Endoscopy, Enterprise imaging, Enterprise imaging platform, Enterprise Image Repository, Enterprise PACS, EXIF, FHIR, Image file format, Integrating the Healthcare Enterprise (IHE), Image compression, Image distribution, Interoperability, Medical image sharing, Metadata, Migration, Multimedia, PACS, PACS implementation, Photography, Vendor neutral archive (VNA), Visible light, Whole slide imaging, XDS, XDS-I

## Abstract

This white paper explores the technical challenges and solutions for acquiring (capturing) and managing enterprise images, particularly those involving visible light applications. The types of acquisition devices used for various general-purpose photography and specialized applications including dermatology, endoscopy, and anatomic pathology are reviewed. The formats and standards used, and the associated metadata requirements and communication protocols for transfer and workflow are considered. Particular emphasis is placed on the importance of metadata capture in both order- and encounter-based workflow. The benefits of using DICOM to provide a standard means of recording and accessing both metadata and image and video data are considered, as is the role of IHE and FHIR.

## Scope

This paper is one of a series of white papers on Enterprise Imaging developed by HIMSS-SIIM workgroups [1–6]. It focuses on describing and solving the technical challenges of enterprise imaging, and does not dwell on the rationale for enterprise imaging except to the extent that the use cases illustrate the technology.

The primary area of focus is the inclusion of visible light imaging into an enterprise imaging strategy together with radiology and cardiology, such as might be found in clinical (as opposed to research) use in a typical large adult or pediatric patient care facility, or an integrated group of ambulatory facilities that share a common infrastructure. It does not address several other areas that might well be considered appropriate candidates for inclusion, but which have highly specific workflow requirements and are reviewed elsewhere, specifically radiotherapy [7] and dentistry [8].

This paper provides insight into the type of devices, formats, and standards involved, along with the associated metadata needs and communication protocols for transfer. It also reviews alternative methods of managing these images and their associated information (metadata), and the common types of systems used.

The companion white paper on enterprise viewing [2] provides a high level overview of the requirements, technical challenges, and solutions for query and retrieval of images for display. This paper elaborates on the available standard protocols and formats that support viewing use cases, but does not address the tools necessary for user interaction or specific to a particular clinical use, nor such issues as color consistency, speed and responsiveness, appropriate choice of display hardware, or the importance of the viewing environment, whether for desktop or mobile devices.

In the interest of interoperability, throughout this paper a strong emphasis is placed on the use of standards, both formal and de facto. Interoperability between systems from different vendors is a prerequisite for successful Enterprise Imaging. The use of closed, proprietary systems that lack standard open interfaces, formats, and protocols is eschewed.

There are different approaches, patterns, and practices for acquiring, storing, and displaying enterprise imaging data. These need to be reviewed and understood before making a choice for a particular deployment. Considerations include the installed base of equipment, software, formats and standards, the relative priority of different use cases and the mixture of medical specialties, existing workflow practices, the size of the enterprise, and extramural integration partners. Given the diversity of users, a mixture of different approaches is often needed and centralized components may have to support several alternative standards in order to be successful. Accordingly, this white paper attempts to be descriptive rather than prescriptive in its exploration of the possibilities.

The use of APIs to access image data and metadata will also be considered, as an alternative to handling images (or sets of related images) using a document-oriented paradigm.

A comprehensive set of references is provided, since one white paper cannot do justice to the entire field; the lessons learned by both early adopters and mature practitioners are expected to be useful to readers.

## Architectural Considerations for Enterprise Image Management

This section makes reference to various source devices, standards, protocols, services, and formats that will be described in more detail later in the paper.

An enterprise imaging strategy is needed for acquiring, storing, indexing, and viewing images from many sources.

Historically, radiology and cardiology have managed images at a departmental level, though have used PACS and related systems to provide access to them throughout the enterprise, and to some extent, beyond the enterprise. Other specialties, particularly those creating visible light (VL) images, have until recently been less concerned with providing access outside the department. Since radiology and cardiology PACS are already present in the vast majority of hospitals, the infrastructure, backup, disaster recovery mechanisms, and governance policies can be shared with other departments using VL imaging for whatever purpose [9–11].

The technological challenges of enterprise imaging are essentially those of how to leverage, consolidate, or replace existing PACS infrastructures with systems using standards and technologies that provide access throughout the infrastructure for all imaging specialties, using the most efficient and reliable mechanisms. The term “enterprise imaging” was initially used to describe extending support for traditional radiology and cardiology modalities to the enterprise [12–14], but today, it is recognized to include all potential sources of images, what early pioneers referred to as “multimedia imaging” [15, 16].

The enterprise imaging strategy is the roadmap with governance that defines the approach, the supported services, data formats, and acquisition methods, and provides the underlying infrastructure for storing, indexing, and accessing the images when and where they are needed.

There is no single correct strategy, but there are some common themes. The first is that there needs to be an enterprise imaging platform of some sort.

### Enterprise Imaging Platform

An enterprise imaging platform provides the reliable infrastructure, and integration points, on which the enterprise imaging strategies and initiatives can be based. A high level view of a generic enterprise imaging platform is illustrated graphically in an accompanying white paper [3]. At the center of an enterprise imaging platform is an Enterprise Image Repository (EIR), which provides standards-based image archive infrastructure and services for storing and retrieving DICOM and non-DICOM clinical imaging content. It includes an index of the images and content in the archive, along with related meta-information that was recorded when the images were initially stored. It also usually includes a viewer for viewing the imaging content in the archive.

An Enterprise Image Repository may be a single system or a system that provides a federated view of multiple underlying components. An Enterprise Image Repository may or may not be implemented (or branded) as a so-called Vendor neutral archive (VNA). To the extent that a VNA may have features that may not be required for this application, and to the extent that the requirements for an Enterprise Image Repository may be satisfied by other systems that do not claim to be VNAs, the Enterprise Image Repository terminology is used here. The word “repository” is used in a general sense and should not be confused with the IHE Document Repository actor (indeed in IHE XDS-I, images are served by an IHE Imaging Document Source actor, and the Document Repository stores only the manifest of images). The term also serves to emphasize the enterprise-wide utility of the system.

Surrounding this core infrastructure are standards-based interfaces and services that enable and support various image acquisition workflows or query/retrieval of the images with related content.

These standards-based services include a modality work list service for communicating accurate patient demographic and procedure information for order-based workflow, a reliable and highly available archival service for maintaining the images securely over time, and a query/retrieval service to find and retrieve imaging studies using standards-based interfaces. The term “study” rather than “exam” is used in this paper, for consistency with DICOM, and to avoid confusion with billing conventions that may dictate what is declared to be an “exam” in a particular jurisdiction.

Standards-based services may also include other services such as image routing and coercion, DICOM wrapping, EHR/EMR integration, auditing and access control, image exchange, and retention management services. The term “coercion” is used in this setting to describe recoding of one value into another, usually for the purpose of using a canonical standard value, e.g., to convert a patient’s name into the standard format that is used in the master patient index, to correct a misspelling, or to convert a proprietary code into a standard code. Coercion is sometimes also referred to as “tag morphing” in a VNA context, though that implies a certain ad hoc approach and a lack of discipline compared to what is usually construed as “coercion.”

These standards-based interfaces are usually based on DICOM, DICOMweb, or XDS-I.b, allowing acquisition devices that support these interfaces to store their images, with meta-information, into the image repository. Image acquisition devices that are supported include DICOM imaging systems from a variety of different departments, point-of-care acquisition modalities, mobile device photographic or video apps, digital recording systems in procedure rooms, image exchange gateways, and software designed to import medical photos saved on a disk, or received as secure attachments (e.g., via patient portal or email).

### Enterprise Content Management (ECM) Systems

It is common for EMR systems to have an integrated ECM system to manage, among other data types, scanned documents, such as forms and historical paper records. These systems can generally store files and associate them with the patient identity within the EMR, and may also allow the stored files to be associated with orders and/or encounter information. The primary users are clerical and administrative personnel.

The ECM system will typically include a document viewer and have a method to notify the EMR of the availability of content to present information and links to the EMR user to access the content.

It can be a common discussion, as part of an organization’s Enterprise Imaging strategy development process, to assess the role of the ECM system. In some cases, the question is asked whether this type of system, which is often already in place and staffed with trained personnel, can be used as the Enterprise Image Repository.

Certainly, there are types of Enterprise Imaging content that resemble documents more than medical images. In some cases, departmental resulting applications that produce documents will store their multi-media output to the ECM system.

Before considering an ECM system as your primary Enterprise Image Repository, a detailed assessment of its technical capabilities, including its use of healthcare and general IT standards in its APIs, is highly recommended. While integration with document scanners is common, integration with other devices, including consumer cameras and diagnostic imaging modalities, is often less common or immature, compared to other applications on the market. For example, to the extent that a particular ECM cannot adequately replace the functions or DICOM standard interfaces of a radiology or cardiology PACS, it may not be sufficient alone as an Enterprise Image Repository; hence, an additional archive system from a different vendor, separate from the ECM or providing more functionality on top of it, may be necessary to produce a complete Enterprise Imaging Platform.

### Integration with the Departmental Imaging Systems

The Enterprise Imaging Platform integrates into the departmental workflows to reliably archive the images that are generated by the department and make them accessible wherever they are needed in the enterprise. This is a significant service for both departments and for the enterprise. Departments do not have to maintain their own archives or historical data. Having the images centrally archived enables clinicians to get a complete view of the patient imaging records, regardless of the department or system that originally generated the images. It also provides a way for other departments to access images they need for their care (e.g., orthopedic templating, surgical planning and guidance, pre-fetching historical studies, creating 3D views, etc.).

Many medical departments generate or use medical images in their practice, whether for diagnostic purposes, for documenting a procedure or finding, or for surgical planning and guidance. The HIMSS-SIIM foundations white paper categorizes the different use cases into diagnostic imaging, procedural imaging and evidence imaging, as well as describing image-based reports [3]. Further detail is considered in the “Source Devices” section of this paper. For the purpose of considering the types of images that departments create, and how to integrate them with the Enterprise Imaging Platform, in this section we will broadly categorize them into groups based on the acquisition pattern.

1. *Departments that use image acquisition devices that natively work with DICOM images and protocols*.
  Radiology, cardiology, dental, ophthalmology, as well as many departments that use ultrasound devices are among those included. These departmental imaging systems include Picture Archive Communication Systems (PACS) and a variety of modalities (CT, MR, US, CR, DX, MG, XA, etc.). DICOM Modality Work List services provide accurate patient demographic and procedure information to the modality, and well-defined interfaces for storing to, querying for, and retrieving images from the Enterprise Imaging Repository. The image objects are well defined and include the meta-information (patient, study, image, etc.) in “header” attributes that are included in the image file.
2. *Departments that use endoscopes, microscopes, and digital cameras*.
  These imaging sources are part of a family of “camera-based” sources that produce “visible light” images (photos or video). Many endoscopes, microscopes, and cameras do not natively create DICOM images, although some include software that can, and third party tools are available. For older analog devices, a “digital capture system” can acquire both “still” images and “video” clips from the endoscope, microscope, or camera’s video signal, though most modern devices use digital sensors and create and store digital images directly. The acquired images and videos can be saved in either DICOM or consumer camera (non-DICOM) formats (JPEG, MPEG, Tagged Image File Format (TIFF), DNG or Camera RAW, etc.). To obtain accurate patient demographic and order information, for order-based workflow the DICOM work list service can be used, and for encounter-based workflow the DICOM query services, a record of HL7 ADT messages, or IHE PIX and PDQ profiles can be used.
3. *Medical photos or videos that were previously captured, or have just been captured, and saved as consumer camera format files, which need to be imported into the Enterprise Imaging Platform*.
  For operator convenience, particularly on mobile devices like phones and tablets, the import and capture applications may be combined into a single application and made a single workflow step, since most devices provide a camera API that is accessible to healthcare-specific applications.
  Like images from endoscopes, microscopes, and digital cameras, these images are generally visible light images (photos or videos) and are usually captured in their consumer camera jpeg, mpeg, or other video format. The images may have been recorded by a separate mobile photo app, imported via a patient portal as secure attachments, or created by some other departmental system or workflow. Import software can be used to associate the images with the correct patient and to provide other descriptive metadata (date/time, anatomy, procedure, indication, etc.); some or all of this metadata may be available from the context in which import application is running (e.g., if the patient is selected in the EHR and the application is synchronized with the EHR); the rest may need to be manually entered by the operator.
  If the import software and the Enterprise Imaging Repository support DICOM, the images can be “wrapped” (encapsulated) in DICOM and the metadata encoded in the DICOM “header”, then stored in the Enterprise Imaging Platform as DICOM objects. Otherwise, the images can be imported in their consumer camera JPEG, MPEG, or other format and the meta information loaded along with the images. If the Enterprise Imaging Repository also supports IHE XDS-I, it may make the DICOM images available using XDS-I transactions and may also serve as an Imaging Document Source and register a manifest of the images in another system acting as a Document Registry (perhaps outside the enterprise).
  If the import software and the Enterprise Imaging Repository support IHE XDS, these native image objects can be treated as “documents” and the XDS transactions used to store the images in a document repository, and record their metadata in the document registry.
4. *Image exchange and sharing: exporting and importing outside studies (foreign exam management)*.
  The Enterprise Imaging Platform is a natural place to import and manage outside studies, and make them available for the departments to use in their respective workflows. An export service is also needed to enable image sharing with other facilities and providers, as well as to give patients a copy of their own record.
  Whether the images were carried in by the patient or received by mail on a CD or DVD and imported manually or automatically, or imported via an electronic image exchange, an import service can be used to associate the images with the correct patient. If necessary, orders can be created to enable processing the outside studies in internal systems (e.g., PACS, EMR). IHE defines an Import Reconciliation Workflow (IRWF) profile that standardizes the transactions involved in both scheduled and unscheduled workflow [17].
  Once they are imported into the Enterprise Imaging Platform, they can be viewed in the enterprise image viewers or pre-fetched to a departmental PACS for more detailed analysis and processing.
  The use of a standard format for both importing and exporting is vital. The IHE Portable Data for Imaging (PDI) format [18] specifies the use of DICOM standard media application profiles, which includes DICOM files and a directory of patient information (the DICOMDIR file). Import software needs to be able to read and process DICOM files, but may also be able to cope in the absence of a DICOMDIR, as well as read the proprietary formats of some older PACS equipment. Of course, a facility should only export standard IHE PDI DICOM media to prevent interoperability problems. The AMA recommended standard of practice is that the “all medical imaging data distributed should be a complete set of images of diagnostic quality in compliance with those found in the IHE PDI (Portable Data for Imaging) Integration Profile” [19, 20].
  The use of Internet-based services for image exchange and sharing is strongly recommended, and is the subject of a separate white paper [4].

## Source Devices

Acquisition devices vary among specialties, although many devices may be shared between specialties, as may occur with a hybrid device used for radiology-performed ultrasound and cardiology-performed echocardiograms. The same device type may be used to acquire images across varied specialties. For example, video capture devices are used to acquire video and still images through endoscopes, cystoscopes, microscopes, laryngoscopes, and bronchoscopes.

Traditionally, classification of medical imaging devices relative to purpose has been diagnostic or non-diagnostic and relative to image format has been defined generically as DICOM or non-DICOM. The FDA regulates medical devices, including imaging devices, based upon classification of risk to the patient and user. Medical software that is added to a consumer device, or which is used to process or view it, may bring it under regulatory jurisdiction.

Other specialties such as ophthalmology, which have historically relied on film photography or manually recorded observations, have made the transition to digital imaging. They are encountering the technical challenges associated with acquisition, storage, and distribution. Just like radiology and cardiology, they are faced with an ever-increasing volume of images acquired. Additional challenges arise from non-traditional workflows, the advent of inexpensive and readily available devices and device apps that capture and create images, and the recognition that these images are useful for multi-disciplinary purposes such as case conferences and consultation.

The technical challenges specific to the different classes of diagnostic imaging, procedural imaging, evidence imaging, and image-based clinical reports described in the accompanying HIMSS-SIIM foundations white paper [3] will be considered here.

The question of intent (diagnostic, procedural, evidentiary, or reporting) has an impact on various technical factors, including the following:

- The fidelity of preserving the original data and the metadata that describes the context of acquisition,
- The manner in which the images are compressed during and after acquisition, for distribution and viewing for interpretation or review, and for archiving,
- The suitability of devices and software used for display and
- The viewing environment (particularly in terms of ambient light).

These technical characteristics are affected by the use case, whether it be interpretation by a dedicated imaging specialist (such as a radiologist), the clinician making decisions about patient care influenced by what is seen on the images (such as a neurosurgeon), the use as a prior for comparison (e.g., last year’s screening mammogram), or retrospective review in the event of an unfortunate outcome (such as by an expert witness during litigation).

### Diagnostic Imaging

Diagnostic imaging, as it relates to radiology and most cardiology acquisition devices, is fairly refined in workflow and delivery of images, most providing capabilities to store raw data according to DICOM standards. Some ECG systems, however, maintain raw data in proprietary formats, often limiting delivery to only a post-processed image of the waveform or monitoring strip.

A majority of ophthalmic imaging devices also continue to trend towards storage of raw data in proprietary formats, with progression analysis and other data analysis achievable only through software analysis programs developed by the respective device vendor. Although most ophthalmic imaging device vendors provide at least a DICOM post-processed image or graphical report option, some smaller vendors may only provide post-processed images in PDF, TIFF, Extensible Markup Language (XML), or other format. In these situations, a central archive or departmental PACS must provide OCR capabilities or XML processing. In ophthalmology, manual entry of patient and exam information is prevalent due to lack of DICOM MWL conformance. Often, the device vendors provide a patient or exam schedule option through their own proprietary systems with HL7 integration of an orders and/or ADT feed or PDQ to an EHR.

Other diagnostic imaging specialties such as OB/GYN may also be presented with challenges based upon lack of vendor conformance with standards such as DICOM Q/R as an SCP, ultimately being forced to choose between refined imaging and resulting workflows or interoperability.

### Procedural Imaging

As defined in the HIMSS SIIM foundations white paper [3], procedural imaging is similar to evidentiary imaging in most often being the “exhaust of the visit” and not the reason for the visit. Technically, both share encounters-based workflow challenges.

Although procedural imaging more often includes traditionally DICOM compliant modalities like CT, fluoroscopy, and ultrasound, the technical challenges include imaging spanning both DICOM and non-DICOM imaging. In surgery, for example, a cholecystectomy may include portable X-rays, ultrasound, and scope video and still images. Additional EMR build challenges in linking all imaging to the same surgical procedure may occur if the imaging performed by radiology is not able to relay or reference the surgical encounter number. Further issues are noted with image display and relativity through an enterprise viewer when the scope video and stills are not stored as DICOM objects and the viewer is unable to display non-DICOM images and multimedia or provide reference as a combined image record.

### Evidentiary Imaging

When considering procedural imaging workflows and device integration, video and clinical multimedia capture, storage, and distribution present numerous technical challenges.

Scopes are found across multiple specialties with each providing very specific abilities to identify diseases, disorders, and pathologies specific to the organ/anatomy being evaluated. A scope alone can provide the clinician the eyes to see within the invasive anatomy, but does not provide the ability to capture what is being seen, thereby requiring a method of capturing video, stills, and even audio. With the advent of handheld devices/smartphones combined with limited capture systems readily available in most specialties throughout most healthcare organizations, providers seeking methods to capture and store can easily find smartphone adapters, lenses, and apps. This has created security, privacy, and availability concerns for captured content.

### Image-Based Clinical Reports

Image-based clinical reports are often produced as post-processed documents detailing analysis and providing graphs, diagrams, or images associated with or providing a graphical representation of the analysis. Devices and systems frequently generating these reports can be found in numerous specialties including neurology, audiology, speech, and ophthalmology.

Often the data compiled to create the analysis remains available only within the acquiring device or in a centralized system able to translate and analyze the data, from one or more vendor-manufactured devices, providing an output including graphical representation of the data, system-generated interpretive analysis, and with or without embedded images. Variances in the ability of non-vendor systems to translate the data from other vendor devices and the capabilities of a device to share data factor into how and what can be stored from an enterprise imaging perspective. Distinctly differentt in acquisition methods can be used across vendor products of the same device type, which would require multiple variables to attain the same output.

Organizations looking to store all data associated with the generation of an image-based report within a single logical environment for availability through a single application may need to reconsider their strategy, as this is currently difficult to achieve. Also of note, workflow will continue to be impacted by image-based clinical report generating devices unable to transmit data electronically, often resulting in a manual print-scan-upload workflow. Many challenges exist for those in the quest of achieving standard data exchange in this area.

In this section, the intent-based classification will be considered for each type of data source.

Diagnostic imaging includes common radiology and cardiology modalities and further incorporates most ophthalmology imaging techniques such as optical coherence tomography (OCT) and ophthalmic visual field (OPV) images.

With the advent and increasing usage of point of care imaging, ultrasound imaging is prevalent within non-traditional specialties. Bladder scans and line placements often acquire ultrasound images to document rather than diagnose, and in these instances can be considered in the category of evidence imaging.

Fluoroscopy is also being increasingly used as documentary evidence and across specialties outside of radiology and cardiology. An orthopedic surgeon may use fluoroscopy to quickly check casting, emergency medicine and hospitalists may use it to check tube placements, etc. Mini c-arms are often owned by varied specialties hospital wide including pain medicine and surgery.

Clinical photos taken with a digital camera or smartphone and not tied to a device providing further analytical capabilities, such as ophthalmic photography (OP) devices, used for retinal imaging, or dermatology images acquired with a dermatoscope and smartphone, are generally considered evidence based. Although one may contend that when used to evaluate a wound or neoplasm over time, the photos are better categorized as diagnostic in intent.

Departments such as dentistry, oral and faciomaxillary surgery, anatomic pathology, dermatology, gastrointestinal and surgical endoscopy, plastic reconstructive and cosmetic surgery, and others use VL in their care workflow. Today these images are often not available through the EMR and may not be routinely or reliably digitally archived. However, in many cases, these are “informally retained” on individual cameras, PCs, and removable media such as CD/DVD/USB. Informal retention does not make these images broadly accessible, and may also perniciously provide problems for HIPAA compliance due to inadvertent disclosure and legal risks of discovery or discovery non-compliance. For example, the Supreme Court of Ohio has ruled that medical data “that a healthcare provider has decided to keep or preserve in the process of treatment” is part of the medical record and must be disclosed, regardless of where the information is stored. The basis was an ECG monitoring strip not made available to the patient’s family as it was not stored in the medical records department but rather on the ECG device [21]. Extreme caution should be exercised when establishing policies for selectively archiving or distributing image data [22].

There are other good reasons to archive these images. They can be used in a store-and-forward workflow to consult with another specialist, or the archived images can be compared over different time points. Even if these VL images are created in a non-DICOM format such as JPEG or PDF, e.g., with a digital microscope or point-and-shoot camera, these images can be encapsulated in a DICOM Service-Object Pair (SOP) Class object after image capture. The patient demographic info and other metadata are then entered or automatically populated in the header of the new DICOM file containing the DICOM VL object. With a formal management system, the issues of image accessibility, HIPAA compliance, availability for required legal discovery, and eventual destruction can be properly addressed.

### Visible Light Imaging Devices

In this section, devices used for various general purpose and specialist applications that use predominantly visible light (that part of spectrum visible to humans (390–700 nm)) will be described. We focus on visible light devices because they present different challenges compared to conventional radiology and cardiology devices that use ionizing (X-ray, nuclear medicine) and non-ionizing (magnetic resonance, ultrasound) radiation, including the use of different types of sensors, the requirement to support true color (typically RGB) images, different metadata requirements, different acquisition workflow, and different requirements for their use. In considering devices for a specific application or specialty, non-visible light devices will be addressed if relevant.

One consideration that is not addressed in this paper is the matter of color consistency, accurate color rendering, and calibrated acquisition and display devices. That is a specialized topic addressed elsewhere [23].

#### Consumer Devices

Consumer photographic devices such as smartphones, tablets, as well as point and shoot and low cost single lens reflex (SLR) cameras make for inexpensive and apparently easy image capture of both still photographs and video. Additionally, many of these devices open the options for dedicated medical image recording applications. However, there are features of these devices that may insidiously interfere with the medical image acquisition requirements. A full best practices discussion would deserve its own white paper but here are some basics:

- Disable auto white balance—set the white balance to match the lighting used (daylight, fluorescent, incandescent, or by color temperature (e.g., 4000 K). Medical images often have predominant colors (e.g., all skin for dermatology photos) that will “fool” the white balance
- Use flash if available. This will improve color balance but may adversely add glare that obscures the image
- If using a “hot light” or spotlight or examination lamp, it should be of the same color temperature as the overall room lighting. It is a poor practice to mix incandescent spot light and fluorescent room light leading to localized white balance problems
- Disable red eye reduction and correction. Some forms of correction actually alter the pixel data.
- Disable blemish correction/removal (sometimes called beauty shot or beauty mode). Skin blemishes are often exactly what we want to record and removing them will alter the data or make the image misleading or useless

Staff should enlist the aid of a professional medical photographer to create a cheat sheet of settings for each device and the room environments in which they are used.

Consumer devices are often limited in the range of file formats that they are capable of storing. They may be network connected (e.g., via Wi-Fi or cellular connections) to enable file transfer, but do not usually implement healthcare-specific interoperability standards, though smartphones and tablets may support dedicated healthcare image and video capture applications.

#### Medical Photography Devices

Many health care enterprises have a dedicated medical photography department staffed by certified professionals, which provides clinical, educational, and research imaging services for other specialties The history of medical photography long predates the availability of digital cameras [24]. Professional medical photographers need expertise not only in photographic technique but also the relevant anatomical and procedural information [25].

In some cases, high volume service lines utilizing visible light imaging such as ophthalmology have their own dedicated medical photography section. While some specialties and applications may not require photography-trained staff, for other specialties, medical photographers play a critical role in a patient’s care. The sophistication of the photographic techniques used may mean the difference between an image that is usable for follow-up care and one which is strictly evidentiary. Over or underexposure will cause loss of detail that may result in missing important information. Proper white balance, lighting, and flash use are necessary to avoid variation between images, in specialties such as wound care where, for example, documentation of burn color or wound traits over time may be crucial. To reduce or eliminate glare, lighting diffusion techniques may be required. Measurements of objects within a photograph require the presence of a reference object of known size or metadata recording the image acquisition geometry (such as the physical size of the field of view in the same plane as the measured object).

#### Dermatology Devices

Imaging is used in dermatology for comparing lesions and skin conditions over time, discussion with patients, remote review (teledermatology), as well as teaching and research. They are not usually used for primary diagnosis, which is performed visually or by biopsy, except in some uses of teledermatology [26, 27].

Instruments used for recording images are digital cameras and microscopes with digital image output. Medical photography in dermatology is important for following skin conditions over time, rather than relying on subjective, written descriptions and inconsistent memories, as well as for education and publication [28]. Still though, many dermatologists do not use photography for reasons of cost and complexity [29]. To mitigate such issues, the use of low cost, reasonable quality, readily available digital cameras has been encouraged and the importance of correct photographic technique and the identification of the patient, date, and anatomic region emphasized [30]. The presence of identifying information and anatomic landmarks in the images is useful in the absence of formal systems that capture this as explicit metadata, but does not facilitate indexing for retrieval, and does present an issue when de-identification is necessary. I.e., for enterprise distribution of such images, a more sophisticated workflow that enables metadata capture with minimal burden to the user may be necessary.

Other than hand-held photography, specific dermatological devices are available for specific visible light applications, including dermatoscopes for epiluminescence microscopy of individual skin lesions [31], and systems for whole body integumentary photography [32], which can produce digital images. Other modalities than visible light are also used in dermatology, including ultrasound biomicroscopy and OCT [33].

The use of DICOM rather than consumer industry formats like JPEG offers advantages for dermatology, particularly with respect to identification and anatomical location metadata as well as interoperability with other enterprise imaging systems, and standard DICOM Visible Light Information Object Definitions (IODs) may be used for most applications [26, 27].

Legal issues related to the use of digital images in dermatology are non-trivial, but beyond the scope of this paper [34–37].

#### Endoscopy Devices

Endoscopy is not a single discipline, but rather a family of technologies used in many specialties. Every cavity or potential space in the human body is a potential candidate for indirect visualization using a device with a light source and a means of conveying and image. Originally, the technology relied on rigid or flexible light pipes (e.g., fiber optics), but with the advent of chip-based cameras, digital acquisition occurs at the tip of the endoscope (distal sensors) rather than at the eyepiece [38].

Gastrointestinal applications included upper and lower gastrointestinal endoscopy, the latter including both colonoscopy and proctosigmoidoscopy, use in conjunction with radiology, such as endoscopic retrograde cholangiopancreatography (ERCP) and proctosigmoidoscopy. Surgical endoscopy has long been used for cystoscopy and arthroscopy, thoracoscopy and mediastinoscopy, rhinoscopy, laryngoscopy, esophagoscopy, and bronchoscopy, and more recently has become a mainstay in general surgery in the form of laparoscopy. In obstetrics and gynecology, amnioscopy and fetoscopy, colposcopy, hysteroscopy, and falloposcopy are performed. Endoscopes may even operate independently, as in the form of the wireless capsule endoscope (WCE), which is swallowed and passes through the gut passively.

The opportunity to digitally store endoscopy videos has been recognized for several decades, and specific requirements, PACS, and medical record systems have been developed for the purpose [39]. The importance of integration with enterprise systems to assure correct patient identification is recognized [40]. Departmental medical record systems may or may not support capture and archival of images and video [41]. Professional societies have issued specific guidance on imaging systems for their own domain, such as gastrointestinal endoscopy, which address such matters as video recording devices, quality, compression, storage format, and both real-time and store-and-forward tele-endoscopy [42].

A special concern for recording endoscopy video is determining the start and end of a procedure, which can be automated to avoid dependence on error prone manual control or requiring post-procedure editing [43]. WCE applications have additional challenges, given the transit time through the gut, and the need to detect and associate video segments with specific events needs to be addressed [44, 45].

#### Ophthalmology Devices

Many systemic diseases, including tumors and tumor-like conditions, endocrine, metabolic, infective, inflammatory, dysplastic, granulomatous, and demyelinating diseases, have ocular manifestations with imaging findings [46, 47]. The availability and accessibility of imaging performed in ophthalmology is crucial in maintaining a holistic medical record, pertinent to other providers as part of the overall clinical evaluation, and therefore an important part of an enterprise imaging strategy.

Ophthalmic visible light applications have historically made use of film photography. With the conversion to the universal use of professional digital cameras, the availability of specific digital acquisition modalities such as for retinal fundoscopy [48], as well as a long history of tele-ophthalmology applications (such as for diabetic retinopathy screening), digital imaging is now ubiquitous. Compared to film, digital imaging raises specific concerns for quality as well as opportunities for processing [49]. In addition, various image-like applications, such as measurement of visual fields as well as measurements for refractive correction and various surgical applications, can be considered to produce a graphic representation of data in the form of an image or a document that is managed like an image. Ophthalmology-specific image management systems are available, as is EHR integration [50–52].

Most visible light photography ophthalmic imaging device types utilize the concept of field of view (FOV) to establish accuracies in measurement. Various imaging sensor technologies, such as charge coupled device (CCD) or complementary metal–oxide–semiconductor (CMOS), are used by device vendors and provide advantages based upon acquisition light levels or noise factors. Optical acquisition physics vary across device types such as slit lamp, OCT, and visual field depending upon the specific structures of the eye being imaged.

The fluorescein angiography (FA) technique differs from ordinary retinal fundoscopy and requires the intravenous injection of a fluorescent dye, and imaging with blue light as an exciter and capture of the yellow-green light emitted, recorded as a series of monochrome frames.

OCT using near-infrared light and high-frequency ultrasound both provide cross-sectional imaging capabilities, and both types of images need to be supported in addition to conventional photographs.

Similar caveats to those applying to digital dermatology and medical photography apply to digital ophthalmology. In a deployment for Enterprise Imaging, storing the images in DICOM format (encapsulation) will be advisable [53] as is the use of DICOM work lists [54]. However, many ophthalmic imaging device vendors do not provide for raw data for analytics and progression evaluations in DICOM format and only provide a post-processed image or graphical representation of data report in DICOM format. Other vendors only offer a post processed image or report in other file formats such as a PDF or XML, requiring the receiving PACS to offer OCR report functionality or customizable XML data indexing capability.

Similar to radiology device vendors 20 years ago, when MIP/MPR reconstruction of a CT scan required vendor-specific reconstruction software or workstations, many ophthalmic vendors today require vendor-specific software to perform any form of analysis, well illustrated by OCT. The collected OCT raw data, not to be confused with RAW visible light imaging, is not only often proprietary, but there can be a variety of methods to both capture and interpret the data, though a DICOM standard has been developed and is supported by most vendors [55]. While some organizations will restrict device purchases to a specific vendor or two and manage image storage and distribution through the respective vendor’s PACS, other organizations, including those with heavy research participation or employing a best-of-breed approach, are forced to maintain multiple applications for each vendor.

Due to the proprietary nature of some ophthalmic device vendor products, challenges include requiring vendor-specific systems to provide patient or exam schedules to devices from separately distributed ADT or order message feeds. For some vendors, company size and device market cost contribute to additional challenges in support of standards like HL7 or DICOM or general support of functionality like patient or exam schedules.

Quality control processes are essential to provide reproducible, accurate, and precise results. Specifically trained ophthalmic medical photography personnel are necessary, and as with other medical specialties, certifications are available from various professional societies.

#### Anatomical Pathology (Selected Fields and Whole Slide Imaging)

The history of digital pathology is long and convoluted [56] and fraught with intellectual property issues, some of which affect enterprise imaging [57]. Initial efforts to capture and distribute histopathology images were based on use cases involving selected images, e.g., to illustrate the pathology report [58, 59].

By contrast, whole slide imaging (WSI), in which the relevant part of the entire glass microscope slide is scanned at high resolution, is being considered as a potential replacement for use of the microscope for primary interpretation, i.e., virtual microscopy [60–64]. Regulatory issues, uncertainty over image quality and observer performance, and practical productivity and infrastructure issues continue to be a barrier to widespread deployment [65–72].

Slide scanners may be used for single slice scanning, but can also be deployed in automated multi-slide and batch mode systems, processing and scanning 200 slides per hour or more. The images may be stored in several different resolutions for display on a computer monitor at different magnifications. Some systems can store multiple focal planes as multilayer objects, which significantly increases the size of the images.

Standardization of the formats and protocols used for WSI and the ability to reuse existing enterprise infrastructure for storage and distribution are regarded as crucial issues, so DICOM Working Group 26 addressed storing digital slides within a PACS archive in DICOM Supplement 145 [63, 73–75]. Despite the availability of the DICOM standard, proprietary formats, many based on TIFF, are in widespread use and interoperability between different systems from different vendors is limited [76]. There are also competing standard image formats developed that are specific to the WSI application, such as OME-TIFF [77, 78] from the Open Microscopy Environment [79]. Various commercial and open source platforms exist to convert proprietary formats into common formats exist, to enable vendor-neutral viewing and image processing [80–83]. The use of JPEG 2000, with its inherently multi-resolution structure in the wavelet domain, seems like a natural representation for WSI, but it is not used by vendors, who prefer traditional JPEG compression of tiles, and has seen limited application in virtual microscopy viewers [84, 85] with or without use of the associated of the standard JPEG Interactive Protocol (JPIP) to retrieve selected regions for interactive viewing [86].

As a practical matter, whole slide images are so large, and the manner in which they are viewed is so specific (i.e., using a “virtual microscopy” navigation paradigm that is more like a whole earth geographical system such as Microsoft Terraserver [87] or Google Earth [88]), that dedicated systems may be required, and it may be inappropriate to burden an Enterprise Imaging Platform with this class of images. While data volumes for WSI might be tractable if used only for remote consultations, case conferences, and teaching, if all of the slide output of a busy pathology laboratory were to be digitized, the archived data volume might exceed that of all other enterprise imaging applications combined. For example, one site describes producing 380,000 slides per year (1500 slides per working day) [89], which at 0.4 GB per slide (after lossy compression, expect an average range of 200–650 MB [69]), is approximately 150 TB per year. Another site, a large tertiary referral center generates 1.4 million slides per year, which at the 0.4 GB per slide estimated file size would be 560 TB per year, compared with the same facility’s current volume of images from all existing DICOM sources of 150 TB JPEG losslessly compressed (280 TB/year uncompressed; 80 % is 2.1 million radiology studies per year, and remainder cardiology, obstetrics and gynecology, etc.). Scalability is not only a requirement for archiving and distribution, but also for processing [90].

### Waveforms and Other Multimedia Objects

The HIMSS-SIIM foundation white paper [3] defines Enterprise Imaging as:

“The management of all clinically relevant content, including imaging and multimedia, for the purposes of enhancing the electronic health record through a set of strategies and initiatives designed and implemented across the healthcare enterprise. These strategies and initiatives are based on departmental and specialty workflows for all clinical imaging content, and include methods for capture, indexing, management, storage, access for retrieval, viewing, exchange and analytics.”

Medical workflows across many departments capture and create a variety of types of “multimedia” information that is important to preserve, correlate with the images, and make accessible via the medical record. This multimedia content includes waveforms, audio or video clips, as well as other forms of graphical content that summarize imaging results with the results from other medical procedures and tests. Examples of this are present in many specialties including Ophthalmology, Cardiology Neurology, OB, and GI.

Graphical “report-style” results from various medical departments are increasingly being created and saved as PDF objects. These can include embedded images that show key findings, graphical diagrams that show the area of interest, or other measurement or test result information that correlates with the images.

Another example of related multimedia content includes time-based waveforms such as those produced by ECG or EEG devices. These may be treated as documents or image-like objects. Waveforms may be recorded and stored in a raw or processed form that requires an application to display them, or in some human-readable rendered form (like a PDF or screenshot). Like images, waveforms too can be classified as both evidence and diagnostic. Waveforms are the graphical representation of discrete data points but may be used as the sole basis of interpretation when other tools for analysis of discrete data points are not available or routinely incorporated within the interpretation protocol.

Most types of multimedia content, including waveforms, PDF reports, MPEG video clips, and JPEG photos, can be DICOM wrapped and stored as DICOM objects or they can be treated as a native document type (e.g., PDF, JPEG, MPEG, etc.) and saved in system that can manage them as native objects. What is important is to consider how this information will be managed, correlated, accessed, and viewed by physicians and patients. Related images and multimedia content should be easily discoverable and shown together in a natural way.

## Standards, File Formats, Protocols, and Profiles

Almost any image file, regardless of the format in which it was originally acquired, can be encapsulated in DICOM and stored as a DICOM file or transmitted using a DICOM protocol. DICOM has a long history [91] of adding specialty-specific information objects [92], in addition to having general purpose secondary capture as well as visible light photography [59] and video object support [93]. Throughout this paper, references to DICOM Supplements are for historical purposes only. Implementers should take care to consult the current edition of the standard [94], since corrections may have been made since the publication of the supplements, which are not themselves maintained.

Yet some organizations may choose to maintain some images in their native format. In this section, the rationale for payload and protocol choices will be examined.

Non-DICOM and DICOM image files may include still images, dynamic image sequences, waveforms, video, audio, and multimedia documents. Non-DICOM, or native, images may be acquired and maintained as standard digital image and multimedia formats such as JPEG, TIFF, WAV, and MPEG4. Some vendors choosing to provide acquisition in proprietary formats may still deliver post-processed images in standard file formats.

Any enterprise imaging solution, by definition, is all-inclusive, so support for DICOM images from radiology and cardiology is mandatory, for acquisition, storage, distribution, and display. This applies to the entire Enterprise Imaging Platform, including repositories and viewers. I.e., nobody is suggesting that more than 30 years of progress since the first ACR-NEMA standard [95] (which became DICOM) be discarded. The question is, should all other images also be stored as DICOM, and communicated as DICOM, or are other formats and protocols appropriate and do they need to be supported in an Enterprise Imaging Platform? I.e., is a pure DICOM solution sufficient, or is a hybrid approach that supports DICOM and non-DICOM payloads necessary or desirable?

To help answer that question, the following sections examine the importance of collecting relevant and accurate metadata, the methods of metadata acquisition, use and management, and the relationship with the choice of compression, file format (non-DICOM and DICOM), and communications protocols.

### Metadata

The first key question is that of metadata. Of particular importance is metadata related to identifying the patient and date and time of acquisition, without which the images would be useless for clinical or evidentiary purposes. To be most useful, all aspects of study context, including modality and clinical specialty related acquisition data are required. Ideally, this metadata should be standardized, structured, and coded [96, 97].

There are two patterns for associating metadata with images, to store the metadata:

- Within the image files, as DICOM does, or
- Separately in a system that associates it with the image files.

DICOM provides support for encoding both generic identification and modality and specialty-specific acquisition context for all enterprise imaging modalities. In theory, DICOM-like metadata can also be added to other image file formats like JPEG or TIFF, though this is rarely done for medical metadata (but is common for photographic metadata, such as in the Exchangeable Image File Format (EXIF) extension to JPEG). Other alternatives include encapsulating the image in a different standard format, such as HL7 Clinical Document Architecture (CDA), as is defined by the IHE Scanned Document (XDS-SD) profile, so that the metadata remains directly associated with the image. Strictly speaking, the XDS-SD profile defines a limited set of media types that may be encapsulated, but the approach can be generalized.

Standard protocols also exist for submitting metadata separately, and in particular the IHE XDS metadata can be populated, if images in their acquired format are submitted as documents (essentially as opaque blobs). XDS metadata is a small subset of what is available in DICOM metadata. While this provides a standard means of submitting metadata, there is no standard means of retrieving a serialized form of the metadata together with the image from an XDS system. The IHE Cross-enterprise Document Reliable Interchange (XDR) profile provides a standard transaction for transmitting metadata and the documents or images with which they are associated, and there is a variant specifically for imaging, XDR-I, but there is no standard mechanism to initiate such a transaction from an XDS system. Both XDR and XDR-I should be considered as potential image submission mechanisms, however.

Other types of metadata that may be important include the following:

- Clinical metadata, describing such things as the anatomical location, functional conditions present during acquisition, and substance administration
- Technique metadata such as photographic technique and camera settings and characteristics (e.g., shutter speed, aperture, ISO sensitivity setting, lens focal length), which may be important for quality control, repeating the same technique for follow-up, and for research or developing future advances in imaging, including computer-aided detection

### Workflow and Sources of Reliable Metadata

Digital acquisition of imaging data may happen at the moment of the patient encounter or be performed retrospectively by digitizing analog or physical media. A lesson learned in radiology and cardiovascular medical imaging since the first availability of commercial PACS in the early 1990s is that capture and association with metadata at the time of the procedure is preferable.

Regardless of how the images are acquired and stored and indexed, whether using DICOM storage services or some other mechanism, reliable metadata must be obtained. The wide variety of patterns of workflow and image acquisition methods for different equipment and specialties requires different combinations of transactions in an enterprise imaging environment [10].

The acquisition process can use pre-prepared information in the case of order-based workflow. IHE Scheduled Workflow (SWF), though designed for traditional DICOM-based radiology and cardiology workflows, has been used for many other types of image capture, such as IHE Eye Care Workflow [98]. IHE SWF is globally implemented in hundreds if not thousands of hospitals. The intent of these standards is to assure that the images are associated with not only the correct patient but also the correct order (such as by providing the accession number to be recorded in the meta data), in addition to providing reliable contextual information (such as the procedure description, codes, and reasons for performing the study). A challenge for image capture with mobile devices is that there is as yet no lightweight (e.g., RESTful) equivalent of the DICOM Modality Work List (MWL) C-FIND service, though in practice most acquisition devices that are used in an order-based workflow are DICOM capable in this respect (if for no other reason than VA procurement requirements have “encouraged” this [99–101]; these apply to “all VA and DoD purchases of radiology, dental, ophthalmology and optometry, cardiology, pathology, endoscopy, dermatology, and all other DICOM-compliant digital acquisition modalities and related equipment”).

For order-based workflow, when an imaging study is scheduled, the EMR (or other information system) will automatically populate the imaging device’s modality work list. Through the work list, the imaging device obtains the patient’s demographic data as well as the order information. Patients are expected to be registered and their demographics are obtained by the work list provider using an HL7 V2 ADT message. Selection of the correct work list entry is often automated through the use of barcode scanning of the patient’s wristband or documentation that accompanies them. The operator does not have to re-enter the patient demographic data or the order information at the imaging device, and a significant source of errors is therefore eliminated. Manual entry of demographic data creates extreme problems in data management and when attempting to compare images recorded in different sessions over time [102, 103]. In order-based workflow, manual demographic entry should be treated as the exception or emergency case not the norm (this is the “unscheduled” case, in IHE terminology). HL7 ADT updates to the archived image demographic data are also required so that the patient demographics do not become stale or out of date over time (IHE Patient Information Reconciliation (PIR) [104]).

Once the acquisition is completed, images are transmitted to the enterprise image repository. The EMR (or other information system) may be automatically notified by a standard mechanism that a new image dataset is available for reading (instance availability notification), which may trigger additional events, such as an update of a reading or review work list to include the new study. This work- and dataflow model applies to all imaging modalities in traditional Imaging departments. When images are produced in other locations such as OR, ED, ICU, CCU, or in patient rooms, the imaging devices in these departments are also connected to the network. In some cases, mobile imaging devices such as a C-arm or portable/mobile X-ray systems are intermittently connected, e.g., with Wi-Fi. The same workflow principles apply.

For encounter-based workflow, the same emphasis on reliable identifying information and the need to mitigate the risks of manual entry apply. In the absence of an order, a Patient Demographics Queries (PDQ) [105] to a reliable source of patient information (such as an EMPI) assists in identifying and validating the existence of a patient at certain location. The IHE Web-based Image Capture (WIC) integration profile [106] was designed to specifically address some of the challenges encountered in capturing images from mobile devices and using applications that depend on a minimal understanding of the necessary standards like DICOM and DICOMweb. In particular, it is intended for encounter-based workflows and specifies the use of PDQm [107], a lightweight RESTful version of PDQ, to obtain reliable demographic information, when the image capture is not performed in the context of another application from which the metadata is already available (e.g., a mobile EMR app). IHE WIC also suggests the use of barcode scanning capability already available within the mobile device to automate and increase the reliability of retrieving the correct identifying information.

However, PDQ only addresses the patient and not the encounter.

In the absence of encounter-specific information or a prospectively generated order, it is challenging for receiving systems to validate incoming images against a specific encounter and associate them with such an encounter. Unless some contextual information is provided by the source system, the metadata supplied may be limited to only such things as the date and time and location of acquisition. The clinical context may already be available from other running applications (and made available through proprietary interfaces or using the Clinical Context Object Workgroup (CCOW) interface) [108]. Or, it may need to be entered or selected by the operator of the image capture application. In any case, it is desirable that identifying information be automatically validated, and descriptive information provided [92, 109].

Difficulty may arise not just in associating the images with the correct encounter but also with triggering the EMR (or other information system) to file a solicited or unsolicited result message or notification against a specific encounter from the enterprise image repository, which may be necessary to trigger downstream activity by some personnel, such as to view or interpret the images. This may require the staff to know by some other means that the patient received the imaging and potentially have to search through a plethora of content to locate it (e.g., by encounter date).

To account for limited data validation performed during acquisition, enterprise image repository systems may need to use other sources of reliable demographics, such as HL7 V2 ADT feeds or an EMPI, and to update (coerce) the patient, visit, and order information in the received image metadata accordingly. Some organizations may also restrict direct queries to EMR and may not have the means to add another software layer for managing transactions, rules, and queues. EMR integration must go beyond patient level availability for meaningful content delivery to providers and the metadata details commonly associated with DICOM image sets are needed to maintain a continuous, multi-disciplinary image record.

When technical capabilities and standards supporting encounter-based workflows are limited or unavailable in a particular organization, it may be necessary to choose order-based workflow instead, and when necessary, artificially generate an order automatically, prospectively or retrospectively. Imaging workflows in various specialties that may benefit from an encounter-based imaging workflow are then forced to choose between clinical conduciveness and cohesive integration. Further functional and technical challenges associated with delivering orders-based and encounter-based workflows are detailed in an accompanying HIMSS-SIIM white paper [5].

### Image Compression and Compressed File Formats

Lossless compression is also known as reversible compression and this is the terminology used by the FDA. The lossless compression methods preserve all of the image information, but the amount of compression achieved is rather modest. Depending on the image, and its noise content or randomness, it may be as low as 2.5:1 or up to 6:1 for radiological images [110].

Lossy compression is also known as irreversible compression. That is, you cannot exactly recreate the original image but instead a “reasonable” replica. Lossy compression methods discard what is determined to be less important information. Typically this means a loss of fine detail (blurring), and with higher compression levels, “blockiness,” “splotchiness,” or bar-type artifacts can appear. Typically lossy compression ratios of digital photographs are in the range of 10–50:1. Videos are typically compressed even more. The amount of compression usable for visible light images may be higher than is reasonably achieved for radiology or cardiology images.

The naming can be confusing as some standards offer both lossless and lossy compression schemes (e.g., JPEG has lossless and lossy schemes, though only the latter is used as a consumer camera or web format).

“Visually lossless” is a term used to describe lossy compression schemes and parameters for which it has been determined that a particular human observer cannot discern a difference. That does not mean however that the actual performance of the human observer for a particular diagnostic task, or quantitative measurements of size or intensity made from the compressed image, or the performance of a machine observer (such as a CAD system) is not affected [111–113].

Diagnostically Acceptable Image Compression (DAIC) is a term used to describe lossy compression schemes and parameters for which it has been determined that for a particular type of image and a particular family of diagnostics tasks, compression does not adversely affect performance [114]. Whether or not lossy compression is appropriate and under what circumstances is controversial for some applications, even forbidden by regulators for mammography [115], left to the determination of the individual user [116, 117], the subject of guidance by specific national professional societies [118–120], or a matter of routine for some applications given the standard of care established using earlier analog methods [121]. For medical devices used for viewing images, the FDA requires that when an image is displayed, it be labeled with a message stating if irreversible compression has been applied and with approximately what compression ratio [122].

#### Compression Lifecycle

Lossless and lossy compression may be applied at different stages in the lifecycle of an image. The image may be acquired in a compressed form, at least from the perspective of the user. That is, a digital camera or an ultrasound device may emit images that have already been lossy compressed, and this may or may not be configurable by the operator. An acquisition device may create uncompressed images but during transmission to the repository, lossless or lossy compression may be used as appropriate to reduce transmission time.

On receipt in a repository, lossless compression is often applied to uncompressed images to save storage space, and already losslessly compressed images may be recompressed with a different, more effective, scheme.

During retrieval from the repository by a viewer, if it is appropriate for the task for which the images are being viewed, lossy compression may be applied to reduce transmission time, or progressive lossy to lossless compression may be used to reduce initial response time.

Over time, as the retention period for images expires, rather than discarding them, they might be lossy compressed or lossy compressed again to a smaller size. Managing different “versions” of the same images that have been compressed in different ways is not without its challenges, especially with respect to the unique identifiers of each image and the maintenance of referential integrity [123].

There may be significant reduction in image quality if an image that is lossy compressed is compressed again in a lossy manner, especially with a different scheme. For example, it is not generally appropriate to decompress lossy JPEG images and re-compress them with JPEG 2000. By definition, losslessly compressed files may be decompressed and recompressed losslessly without any change.

#### Schemes, File Formats, and Containers

Compression schemes are frequently confused with the file formats that use the compression schemes, but it is an important distinction. Many file formats are just the stored form of the bit stream produced by the encoder for a particular compression scheme. Other file formats involve the use of some sort of “header” or “container” structure that encapsulates the compressed bit stream. Some file formats are tied to a single compression scheme or variants of a single scheme. Other formats are containers for an extensible variety of compression schemes. The software or hardware that performs the compression and decompression is referred to as a “codec” (COder DECoder). Even though an extensible container format is supported by a system, there is no guarantee of being able to decompress a particular scheme if the codec used for that particular image is not supported on the particular device that is being used to view the images. This is particularly true for the plethora of proprietary video codecs that are encountered in the consumer domain.

In general, the selection of a standard compression scheme encoded in a standard container format is preferable to the use of exotic or proprietary methods. Otherwise, interoperability is compromised. This is particularly important for archives, since the hardware or software to decompress non-standard schemes may not be available in the future.

Container formats not only define the type of compression scheme used, but may also be capable of encoding metadata (including data about how the image was acquired). Multimedia container formats that are common in the consumer applications include formats such as AVI (Audio Video Interleaved) and MOV (QuickTime). For professional and scientific applications, the TIFF format is common.

DICOM is itself a multimedia container format, with patient and medically specific metadata fields, and makes use of standard compression schemes as well as allowing for the use of proprietary schemes.

#### Limitation of Photos/Native Format

- There is no standard encoding of patient or acquisition context metadata
- There is no standard means of annotating and marking up images or indicating which images are key images.
- Photographic images do not have inherent pixel spacing calibration available. In order to perform measurements, there will need to be an object of known size incorporated within each image to use for calibration of the measurement tools. This can be a ruler, pre-marked tear off tape (as used in military trauma photos), or other known size object. However even if “known size” objects are included, they need to have their dimensions noted within the image to avoid later confusion or mis-calibration based on a bad assumption about the size of the object.
- Orientation of the image relative to the patient is not recorded (left/right/up/down) in a standard manner.

#### Lossless Schemes and File Formats

Well-known standard lossless compression schemes include JPEG lossless, JPEG-LS, and JPEG 2000. JPEG 2000 will generally offer 1.5× the lossless compression ratio of JPEG for the same image but requires more computation power.

PNG is a file format with its own lossless compression scheme, which is widely used for web browser applications, and was intended to replace the proprietary GIF format.

BMP is an uncompressed file format that offers no compression and is wasteful of storage, but is commonly used in desktop applications.

The TIFF format supports a variety of compression methods, but most commonly uses some type of lossless compression in consumer applications. However, it is generally better to select a different and more widely supported format such as PNG or JPEG lossless if offered.

#### Lossy Schemes and File Formats

Common standard lossy schemes include those in the JPEG and MPEG families. If a scheme is referred to without an explicit statement that it is lossless or lossy, lossy should be assumed (especially in the case of JPEG). Video images are almost always lossy compressed to a bitrate that can be communicated over a specified channel continuously.

Whether or not the degree of compression applied is under the user’s control, and what choices should be made, depend very much on the type of image and the use case. For radiology images, there has been considerable research on the subject and various guidelines developed, though its use remains controversial [114]. Similar literature, if not professional society guidance, exists for the various specialties using visible light images, but is beyond the scope of this article to review.

For hand-held photography with consumer phones and cameras and professional cameras, various quality factors via either a numeric (0–100) scale or with varying textual descriptions may be available through configuration or within a particular image or video capture application. Be sure to consult the device’s documentation, the scientific literature and professional society recommendations, and most importantly test with local typical images to see which settings are acceptable to the users.

For still images, the image pixel matrix size (number of rows and columns) and aspect ratio (columns to rows ratio—e.g., 4:3, 5:4, 16:9) is usually independent of the compression scheme or file format. The more common standard video compression schemes may constrain the matrix size and aspect ratio and other parameters by defining profiles.

Common still frame compression schemes include the following:

- Baseline JPEG (DCT and Huffman coding), which is the most common form of JPEG encountered in digital cameras and on the Web. There are variants that compress higher bit depth images (up to 12 bits) that are supported by DICOM and used for radiology images, but rarely for visible light.
- JPEG 2000, which is a wavelet-based scheme, also supported by DICOM, but rarely encountered in consumer devices or cameras (much to the chagrin of its proponents and designers). JPEG 2000 does not necessarily achieve “better” quality images for the same bitrate as conventional JPEG however. JPEG 2000 has many features that may be attractive for specific medical use cases, including progressive embedded coding, different resolution and quality layers, region of interest coding, and an interactive protocol (JPIP) for retrieving selected regions. DICOM has specific mechanism, the Pixel Data Provider Data URL, and an associated Transfer Syntax, to allow use of JPIP to get access to selected parts of JPEG 2000 compressed pixel data.

Common video compression schemes include the following:

- MPEG2 (Motion Picture Experts Group version 2). Two MPEG2 profiles are supported by DICOM, which were selected on the basis of those most likely to be used for medical images.
- MPEG4/H.264 (AVC). This format is newer than MPEG 2 and offers both higher compression ratios for equivalent image quality compared to MPEG 2 and higher resolution options, including high definition (HD) formats. Two MPEG4 profiles are selected for use DICOM.
- H.265 (HEVC). This scheme offers a 50 % improvement over MPEG4/H.264 (AVC) and is supported by most contemporary smartphones and tablets. DICOM is in the process of adding support for several HEVC profiles.
- MPEG1. This older scheme should not be used for new video acquisition as its support is limited in the field and the image pixel matrix size is very low.
- Motion JPEG. This scheme encodes each frame of a multi-frame image using JPEG and is common in its DICOM variant in medical imaging, such as for X-ray angiography ultrasound, but less common in consumer cameras. It achieves less compression than MPEG2 or 4 for equivalent image quality, since it uses only intra-frame encoding (each frame is independent of all others), compared with MPEG, which uses inter-frame encoding, where each frame is based on the difference between it and the preceding frame.

#### Camera RAW and DNG Files

Photographic image output formats vary across camera manufacturers, models, and smart device apps. Camera RAW format [124] is not a single format, but rather a way to refer to higher quality proprietary image files created by the camera manufacturers. The manufacturer-specific RAW format provides the most information about an image. It is the data recorded by the camera’s sensor with little if any post-processing, and may be thought of as the digital equivalent of an analog photographic negative.

RAW files generally provide greater dynamic range via higher bit depth (9–16 bits per color, but typically 12–14 bits) and can be uncompressed, lossless compressed, or lossy compressed depending on the implementation.

The RAW formats encode metadata about the photo capture including exposure, picture control, and focus. Unless it is being viewed and processed in specialized software, the RAW format requires conversion to a consumer file type such as a JPEG or TIFF. This is often an automatic post-processing event, although most camera manufacturers provide software to manually process the image including white balance, saturation, hue, contrast, sharpening, and compression, and third-party software is available, such as UFRAW.

Many of the proprietary RAW formats are supported by photo and video editors, and this software is regularly updated to support new versions and camera models. Sometimes support for older formats is dropped in recent software, creating a problem for reuse of archived material. RAW formats are not backed as a standard by a standards body such as NEMA, JPEG, or used in the DICOM standards. The RAW formats are intended for advanced image editing including changing color correction, gamma, brightness contrast, and many other parameters prior to producing final output. There is no industry standard way to specify or record metadata for these processing parameters; it is assumed that a final output image in a consumer format will be produced as part of the editing process.

DNG (Digital Negative) format was developed by Adobe as an extension to the TIFF format specifically to address the storage and archiving of raw camera data and avoid the plethora of camera manufacturer-specific RAW image formats, and hence be more interoperable and archivable. It has rich photographic metadata, comparable to EXIF. However, at this time, it is not widely supported as a native format by the major camera vendors (whether SLR or “point and shoot”). This means that a conversion step from RAW to DNG format is required. The advantage of doing this conversion vs. conversion to a consumer format or DICOM is questionable. Depending on the parameters of the conversion, DNG files may be slightly smaller than RAW files. DNG files can maintain the full original RAW data, providing a means for viewing the image without losing the ability to reprocess the image in its original form. Any metadata not recognized by the DNG converter is irreversibly stripped from the original RAW file so care must be taken to examine the true RAW data before any DNG conversion process is initiated.

For final clinical distribution, educational, and publication purposes, consumer formats or DICOM usually suffice, even if the RAW or DNG files archived. Many publications require TIFF images, for example. Currently, DICOM does not have standardized support for encoding the RAW or DNG pixel data or metadata, though theoretically the Raw Data Storage SOP Class could be used for this purpose.

It is interesting to note that the Reuters news agency has banned the use of RAW format in favor of JPEG images produced directly by the camera, ostensibly in the interests of realism rather than artistic manipulation, as well as speed due to the greater transfer time of the much larger RAW images [125]. Both authenticity and speed are important for medical applications too, but need to be balanced against the appropriateness for the use case. JPEG and DICOM images can of course be manipulated too, but protection against this (e.g., with digital signatures or embedded watermarks) has not been a priority, despite there being standards and mechanisms available.

### Native Format Metadata

Photographic technique information may be encoded in a standard manner in JPEG format using EXIF, which adds an application marker segment to the JPEG bit stream that embeds TIFF-formatted metadata [126]. Most professional and consumer camera and mobile devices with integrated cameras produce EXIF. Similar metadata is present in DNG and RAW format images.

### Conversion to DICOM

Though readily available, additional applications, software, or extensions may be required for conversion of any type VL image and videos into DICOM. Ideally, this would be a built-in component of any Enterprise Imaging Platform but in some cases may require additional licenses or fees. The workflow integration issues already discussed are an important feature of conversion applications, since the inclusion of the appropriate metadata in the converted images is essential to their utility.

Some of the advantages of conversion to DICOM include the following:

- They are self-describing image files. All necessary demographics and other metadata are stored as part of the image object. Providers of DICOM network services for query and retrieval (including traditional DICOM and DICOMweb services) can index this metadata, facilitating access, integration, and migration.
- There is an ability to mark up the images. DICOM has the concepts of both key images and presentation states. Key images are to signify the images of importance in the study, which is helpful for downstream users. Presentation states are used to record markup including graphical annotations such as arrows, lines circles, angle measurements, text and numerical values such as from measurements performed on the image, as well as capture display and image processing parameters such as window and level (effectively brightness and contrast), zoom, and pan, which may be reapplied. These presentation state markups are non-destructive (i.e., another decompressed and recompressed copy of the image with the annotations burned in is not required), can be turned on and off by most PACS and enterprise viewers, and additional annotations in new presentation states can be created by additional users.
- A standard set of Transfer Syntaxes (compression formats) is defined, increasing the likelihood of the recipient being able to view the image without reliance on finding obscure or obsolete codecs to decompress and display the image.
- DICOM images can theoretically be stored as dual-personality DICOM and TIFF, without making a second copy, so that non-medical image viewers that support TIFF format such as photo editors and paint programs can display them. In practice, this is rarely implemented, but the DICOM PS3.10 file format [127] contains an unused 128 byte preamble specifically to support this use-case [128] because early ultrasound images were stored by some vendors in TIFF format.
- There are free and commercial viewers capable of displaying DICOM data readily available. These include the ability to compare images captured in different exams/episodes and of different types. Comparison of images over time is a key medical workflow.
- There are defined and proven methods for keeping metadata, including patient and procedure information, consistent with EMR and other information systems.
- There are proven methods for calibrating images for measurements (by including an object of known size in the image), with defined methods for persisting the calibration in DICOM.

#### DICOM Metadata

DICOM defines standard data elements to encode identifying, clinical and technique metadata, as well as the structural elements describing the pixel data itself (matrix size and number of frames, color space, bit depth, type of compression used, etc.).

- The identifying metadata is defined according to a hierarchical information model that includes the patient, study, series, and instance (image) as well as accounting for related information entities such as the visit/encounter, the procedure order, as well as specimen identification. This organization minimizes redundant entry and aids in comparison review of the images. Note that there may only be one component at a given level for a patient or image set (e.g., cardiac angiography images might all be encoded in one series).
- The metadata includes patient demographics (name, medical record number, birth date, sex, etc.) and study or visit level information including the date and time the study started as well as identifiers such as the accession number. The metadata and the definitions of what is in each level of the hierarchy are defined in DICOM within the individual IODs that correspond to SOP classes, which all share the same Composite Information Model of Patient/Study/Series/Instance.
- In DICOM there is the concept of a series, which constitutes a logical set of related images, such as those acquired at the same time using the same equipment (e.g., all the slices of a single MR acquisition). For photographic, video, and audio data, the series distinction may not be useful, but it can be used to indicate a “procedural” break that can be described as “before and after” or “with and without.” This could be, for example, the application of a contrast agent, different lighting conditions (for endoscopy or microscopy), or before and after a clinical event such as excision of a lesion.
- There is additional image level clinical and technique metadata defined in each of the DICOM IODs to support specific applications and which is shared between similar applications (e.g., for slide microscopy or ophthalmology, the optical filters and illumination are described similarly)
- DICOM does not currently define attributes to encode photographic camera technique metadata in DICOM attributes (e.g., shutter speed, ISO setting), though the information may be present in the DICOM encapsulated JPEG bit stream as EXIF.

Note that the discussion of the metadata is meant to be illustrative rather than exhaustive. Refer to the DICOM and IHE standards for a complete discussion.

#### Scope

The demographic metadata for the patient and study can be obtained from a query or manually entered, as has been discussed under the topic of Workflow and Sources of Reliable Metadata. Using a query mechanism dramatically improves the long-term maintainability and comparability of DICOM images as opposed to the use of error-prone, inconsistent, manually entered data. In enterprise imaging, the DICOM images will rarely stand on their own, but will be linked to clinical notes, observations, results, or reports in the EMR, further emphasizing the need for reliable, accurate metadata. In some cases, a “convenience copy” of a report may be kept in DICOM format in the image repository (e.g., to aid in the selection of prior comparison studies), but the image repository is generally not the system of record.

DICOM defines not only standard data elements but also standard value sets for them, many of which can be extended by the site or the users. These may be used to index and retrieve selected images based on standard or site-specific descriptions for the procedure and type of imaging study, the anatomical region (body part), laterality, etc. Usually patients, studies, series, and images may be browsed hierarchically using the more common standard data elements as well as the textual descriptions at each level, and some systems also support free text search of DICOM data elements.

#### IOD and SOP Class

DICOM defines modality and acquisition-specific IODs and corresponding SOP classes, which define the metadata and type of content for specific use cases. Some IODs and SOP classes are intended for specific medical devices (e.g., CT and MR scanners); others are intended for specific applications of visible light imaging (e.g., whole slide imaging) and another set are available for general purpose visible light still-frame and video applications. Finally, there is a family of secondary capture IODs, which are generally applicable, though lacking in visible light-specific descriptive fields, but are widely supported and easily exchanged, even with older PACS systems and viewers.

The entire set of composite image storage IODs and SOP classes share the same patient identifying and order and encounter metadata (i.e., have a common composite context), so they may all be indexed in the Enterprise Imaging Platform in the same way.

When choosing the IOD and SOP class for a particular image capture application, consider both the application-specific metadata (does it have the necessary descriptive fields) as well as the implications for interoperability, e.g., do downstream systems, both internal and external, support the selected SOP class?

Tables 1, 2, 3, and 4 describe some suggested IODs and SOP classes suitable for general purpose and some specific visible light applications in enterprise imaging [129]. Note that when using an MPEG family DICOM Transfer Syntax to encode a video object, the audio channels may be encoded in the MPEG video bit stream [130].

**Table 1:** General purpose DICOM IODs and SOP classes for Enterprise Imaging

Description	Photo (P), video (V), audio (A), document (D), measurements (M)
Secondary capture image storage	P, V
Multi-frame true color secondary capture image storage	V
VL photographic image storage	P
Video photographic image storage	V+/−A
VL microscopic image storage	P
Encapsulated PDF storage	P, D
General audio waveform storage	A

**Table 2:** DICOM IODs and SOP classes for endoscopy applications in Enterprise Imaging

Description	Photo (P), video (V), audio (A), document (D), measurements (M)
VL endoscopic image storage	P
Video endoscopic image storage	V+/−A

**Table 3:** DICOM IODs and SOP classes for ophthalmic applications in Enterprise Imaging

Description	Photo (P), video (V), audio (A), document (D), measurements (M)
Ophthalmic photography 8 bit image storage	P
Ophthalmic photography 16 bit image storage	P
Ophthalmic tomography image storage	P
Ophthalmic visual field static perimetry measurements storage	M
Ophthalmic thickness map storage	M
Wide field ophthalmic photography stereographic projection image storage	P
Wide field ophthalmic photography 3D coordinates image storage	P

**Table 4:** DICOM IODs and SOP classes for anatomic pathology applications in Enterprise Imaging

Description	Photo (P), video (V), audio (A), document (D), measurements (M)
VL microscopic image storage	P
Video microscopic image storage	V+/−A
VL slide-coordinates microscopic image storage	P
VL whole slide microscopy image	P

#### Access to Encapsulated Payload in DICOM Files

An additional consideration, especially for VL images, is whether or not the “payload”, e.g., the compressed still frame or video stream, needs to be accessible downstream.

Maintaining native files may be preferred for organizations utilizing standard multimedia editing software for video and still image manipulation. The extraction of native format from DICOM or XDS-SD CDA objects requires additional software.

DICOM viewers may also be limited in the range of compressed pixel data that is supported. Typical PACS workstations may not support exotic video formats, for example, and even supposedly “universal” enterprise viewers have their limits. Though the repository must support DICOM, the potential cost of additional licenses for DICOM capable devices downstream needs to be considered.

The DICOMweb (WADO-RS) services provide a simple means of gaining access to the encapsulated payload that can be returned in a consumer format media type.

#### Consumer Compression Formats Supported by DICOM

Dedicated medical devices make careful choices about the encoding schemes used to record pixel data, and these are subject to decisions about suitability for the intended use as well as regulatory oversight.

When non-medical devices are used for image capture, there are often multiple image formats to choose from, and the application and the operator may or may not have control over the choice of format and/or parameters that affect image quality.

With each conversion between formats that use lossy (irreversible) compression schemes formats, there may be a further loss in image quality. DICOM provides a choice of standard compression schemes (defined in Transfer Syntaxes), which allow the already compressed data to be encapsulated, rather than transcoded. Conversion of uncompressed and lossless compressed data is always lossless by definition, therefore is harmless. Conversion may be necessary when the receiver does not support a particular compression scheme.

Table 5 summarizes some of the current capabilities of DICOM with respect to encapsulation and conversion of various consumer compressed image formats. The media type that may be requested and returned by the DICOMweb (WADO-RS) services for pixel data extracted as bulk data and separated from the DICOM metadata is listed; note that a media type transfer-syntax parameter may provide further information about the particular compression scheme used when the media type itself is ambiguous [131]. There are also less commonly used MPEG4 stereo video formats available. An exhaustive listing of the transfer syntaxes supported by DICOM is defined in DICOM PS3.5 [130].

**Table 5:** DICOM encapsulation and conversion of various consumer compressed image formats

Format	Type/profile	Convert or encapsulate	DICOM transfer syntax name	DICOM web media type
Uncompressed—e.g., BMP	n/a	None	Explicit VR Little Endian	None—convert to one of the supported lossless media types
JPEG lossless	n/a	Encapsulate	JPEG Lossless, Non-Hierarchical (Process 14) JPEG Lossless, Non-Hierarchical, First-Order Prediction (Process 14 [Selection Value 1])	Image/jpeg
JPEG2K lossless	n/a	Encapsulate	JPEG 2000 Image Compression (Lossless Only)	Image/jp2 or image/jpx
PNG	n/a	Decompress losslessly	None	None—convert to one of the supported lossless media types
TIFF (lossless)	n/a	Decompress losslessly	None	None—convert to one of the supported lossless media types
JPEG lossy	n/a	Encapsulate	JPEG Baseline (Process 1): Default Transfer Syntax for Lossy JPEG 8 Bit Image Compression JPEG Extended (Process 2 & 4): Default Transfer Syntax for Lossy JPEG 12 Bit Image Compression (Process 4 only)	Image/jpeg
JPEG2k lossy	n/a	Encapsulate	JPEG 2000 Image Compression	Image/jp2 or image/jpx
MPEG2	MP@ML <= 720 × 480 NTSC <= 720 × 576 PAL	Encapsulate	MPEG2 Main Profile @ Main Level	Video/mpeg2
MPEG2	MP@HL 1280 × 720 1920 × 1080	Encapsulate	MPEG2 Main Profile @ High Level	Video/mpeg2
MPEG4 / AVC/ H.264	HiP@Level 4.1 1280 × 720 1920 × 1080	Encapsulate	MPEG-4 AVC/H.264 High Profile/Level 4.1 MPEG-4 AVC/H.264 BD-compatible High Profile/Level 4.1	Video/mp4
PDF		Encapsulate	Not applicable	Application/pdf

### Communication Protocol

So far we have considered file formats and divided them into DICOM and non-DICOM categories, more or less equivalent to those with and without embedded metadata. We have further described how DICOM files often contain a compressed pixel data or video payload that needs to be extracted for some applications.

Protocols for communicating theses files and payloads can be classified as protocols for the following:

- Sending, querying, and retrieving DICOM objects
- Sending, querying, and retrieving non-DICOM objects
- Extracting compressed pixel data or video payload from DICOM objects

Other related services exist (such as those for rendering a DICOM image into a consumer format, which often involves windowing and annotating it, as opposed to extracting existing consumer format content as is). These are the URL request (WADO-URI), resource (WADO-RS), and SOAP (WADO-WS) mechanisms for retrieval of rendered images [132]. Since the focus of this white paper is visible light imaging in the enterprise, these rendering services, which are useful particularly for radiology images, will not be discussed further.

Another way of classifying these protocols is to consider whether images are “pushed” or “pulled.” In general, a “push” workflow is used when the recipient of an image is determined in advance and relatively static and when the sender has no robust long-term archive of its own. Image acquisition by a modality or mobile device sending to a central enterprise image repository is a typical example. Users of images, on the other hand, typically “pull” images, which involves first finding them (querying for them) and then retrieving them, which involves making a request to retrieve them and then receiving the retrieved images. Sometimes, the retrieval request and response occur on the same connection, and sometimes they are separated.

Given that there are multiple methods for storing content and metadata to an appropriate enterprise repository, regardless of the features, options, challenges, and advantages of each, serious consideration should be given to requiring support for all of them in a robust Enterprise Imaging Platform.

#### Sending DICOM objects

Traditionally, DICOM has used its own dedicated protocol for sending DICOM objects over the network. This is the DICOM C-STORE operation [133]. It operates over TCP/IP like most other reliable Internet protocols and involves make a “connection” between the sender and recipient. It is most commonly employed within a LAN or via a VPN connection, but can also be used over TLS, though this is not common. Each image object is transferred one at a time, though multiple asynchronous operations can be negotiated (uncommon) and of course multiple connections can be made simultaneously. Each connection begins with a negotiation to assure that the recipient supports the type of object that is being sent and what compression options are available for the transfer. DICOM refers to connections as “associations” using the ISO OSI terminology. Each storage operation is followed by an indication from the receiver to the sender of the success or failure of the transfer (e.g., in case what is received cannot be stored for some reason). Note that using multiple parallel connections is a powerful means of overcoming network latency as a limiter of transfer speed.

More recently, in order to ease the burden for implementations of lightweight sending applications, such as on mobile devices, the DICOMweb STOW-RS (STore Over the Web) has been defined [134]. This allows the sender to use an HTTP POST operation to send one or more DICOM objects, either encoded as ordinary DICOM PS3.10 binary files, or as separate XML or JavaScript Object Notation (JSON) metadata and separate image or video pixel data in various Internet media file formats (e.g., image/jpeg or video/mpeg2).

The STOW-RS service is the basis of the IHE WIC integration profile [106]. WIC attempts to simplify the sender’s task by not only allowing DICOM objects to be sent but also allowing images or videos in a non-DICOM format to be transmitted, along with minimal DICOM metadata in XML or JSON encoding, and places the burden on the server to populate the DICOM metadata that describes the pixel data.

DICOM objects can also be sent using IHE XDS or XDS-I and related XDR and XDR-I mechanism, using the same SOAP-based transaction that is used for transmission of documents in XDS. For XDS-I, an additional requirement is the presence of a manifest of objects, which is a DICOM Key Object Selection (KOS) instance.

The FHIR ImagingStudy resource [135] provides another mechanism for storing DICOM objects, together with a selected set of metadata that can be accessed through the FHIR API (i.e., as elements of the FHIR resource).

#### Querying and Retrieving DICOM Objects

Traditional DICOM provides three services for querying and retrieving DICOM objects:

- C-FIND is the query service, used to locate objects for a particular patient, study, series, or instance, and to obtain information about them, such as might be displayed to the user
- C-MOVE is a retrieval service that requests that studies, series, or instances be sent to another location using a C-STORE operation, i.e., it involves a separate control connection from the connection(s) used to send the images
- C-GET is a retrieval service that requests that studies, series or instances be sent back on the same connection on which the request is made

Of the two retrieval services, C-MOVE is far more widely implemented, but does require that the sender be pre-configured with the IP address or hostname, and port, of the recipient. Hence, it is less suitable for use on a large scale than C-GET for ad hoc requests from non-preconfigured devices from which access could be controlled on the basis of user identity.

DICOMweb also provides query (QIDO-RS) and retrieval (WADO-RS) using the RESTful approach of defining studies, series, instances, and frames as resources specified in the URL of an HTTP GET operation [136]. Both QIDO-RS and WADO-RS allow for the retrieval of XML or JSON metadata, and WADO-RS allows for retrieval of the pixel data (bulk data) in various Internet media file formats (e.g., image/jpeg or video/mpeg2). A traditional C-GET-based DICOM service for retrieving metadata without bulk data is also available but it has not been widely implemented [137].

Both XDS and XDS-I support the query and retrieval of DICOM objects. In the case of XDS, the DICOM images can be indexed in the registry in the same manner as any other object. The XDS-I profile describes the use of an intermediate document, the manifest, which is actually the document stored in the registry. The DICOM instances listed in the manifest can then be retrieved from a separate source, the Imaging Document Source, which may or may not be co-located with the Document Repository or Document Registry. The transactions used to retrieve the images may involve either traditional DICOM C-MOVE operations, DICOM WADO-URI retrieval, or an IHE-specific SOAP-based transaction. As yet, support for WADO-RS has not been added directly to IHE XDS-I, but there is an MHD-I profile in development that makes use of WADO-RS and a FHIR resource-based manifest [138].

In addition, XDS-I extends the limited XDS registry metadata to include imaging-specific metadata including the type of imaging procedure, the modality, and the anatomic region.

The FHIR ImagingStudy resource [135] also provides another mechanism for querying for and retrieving DICOM objects, using the set of metadata that can be accessed through the FHIR API as elements of the FHIR resource. The retrieval of the images identified in the FHIR resource may be through the use of DICOM, WADO-RS (DICOMweb), or WADO-URI using the unique identifiers, or via URLs to WADO-RS or WADO-URI endpoints, though theoretically the images can be included in the resource by inline encoding as media attachments.

#### Sending, Querying, and Retrieving Non-DICOM Objects

IHE XDS supports the storage of objects in any format as documents, though consideration should be given to the use of IHE Scanned Document (XDS-SD) to provide a persistent metadata wrapper.

As described earlier, non-DICOM objects can also be sent using DICOM STOW-RS and IHE WIC, by creating a very minimal set of DICOM metadata in JSON or XML form.

Conversion to DICOM has already been described.

Another standard option that may be considered is the use of the FHIR Media resource [139]. This allows for the provision of content inline (an attachment) or as identified by a URL (perhaps a WADO-RS URL). This resource is related to, but not dependent on, the FHIR ImagingStudy resource [135].

In addition to the use of healthcare-specific systems for use as enterprise image repositories, whether they be dedicated to the purpose or a component of an EMR or other information system, it is also possible to use generic content management or object storage systems for enterprise storage. In general, these make use of proprietary protocols, since there seems to be little standardization of interfaces in that industry. These systems have in common the concepts of separating the payload from the metadata, and supporting indexing and queries on user extensible metadata [140]. This begs the question of the need for a standard for that metadata in order to be interoperable.

There are a variety of proprietary protocols that can be envisioned for ingestion of images. They can be classified based on the interchange type such as follows:

- REST-based
- XML/SOAP-based
- Other methods

However, for each of these there is a near equivalent DICOM or XDS service that could be used instead. It is recommended that when considering the use or creation of a proprietary ingestion method, that strong consideration be given to standards-based transfers and a high bar be placed for approval and implementation of a proprietary method. The content management system vendor should be contacted to ascertain when standards-based methods for ingestion will be provided.

At a minimum, the new system must also provide standards-based methods for storage, retrieval, and viewing in order to avoid a technological “lock-in” to a vendor specific proprietary strategy with no easy escape mechanism. What is particularly problematic is the situation where the images are saved in an opaque proprietary format (database blobs or data files) rather than either standard photographic image formats (e.g., JPEG, JPEG 2000, TIFF) or better yet, DICOM.

Consideration must be given to image exchange and interchange with other institutions as well as eventual migration to a new content management system, VNA or XDS infrastructure [4].

#### DICOM and XDS Security-Related Considerations

For all traditional DICOM operations, though it is possible to communicate user level authentication information when establishing the connection, this is not often done. DICOM supports various types of user authentication, including username and password, Kerberos tokens, and SAML assertions [141].

Recipients can be configured to be “promiscuous” (accept objects or requests from any source) or restricted to accept connections or requests objects from a named set of sources. DICOM uses the ISO OSI terms “Application Entity” (AE) and “Application Entity Title” (AET) to identify endpoints for communication.

When using DICOM over TLS, client and server certificates can be used for more sophisticated control over individual connections and the information used for selective access control and to record in audit trails, as defined in the IHE Audit Trail and Node Authentication (ATNA) integration profile [142].

The DICOMweb transactions can make use of normal HTTP security mechanisms, including those for confidentiality (e.g., TLS) and for user authentication.

IHE defines various profiles related to user authentication and authorization for SOAP and non-SOAP transactions for enterprise [143], cross-enterprise [144], and Internet [145] applications.

### Other Considerations

#### Image and Lifecycle Management and Distributed Consistency

The decision to acquire and store imaging in DICOM or native format may impact image management workflows.

An Enterprise Imaging Platform may or may not implement explicit rule-based lifecycle management, to purge itself of stale content when retention periods expire. Some organizations are satisfied with keeping everything forever, especially in the face of growing rates of image acquisition volume that make the size of historical images a decreasing fraction; others are not. The policy behind such decisions is out of scope for this paper, but the need to communicate in a standard manner to other systems that images have been or are to be deleted for life cycle management purposes may be a consideration.

For DICOM images, IHE defines support for object lifecycle and correction management in the Image Object Change Management (IOCM) profile [146].

Non-DICOM images stored using IHE XDS profiles do not account for many image management needs in a real-world implementation.

#### Migration

Many enterprises have already experienced the challenges of migration of large-scale systems such as PACS, departmental and hospital information systems, and EMRs.

For enterprise systems, the scope of migrations to be considered needs to include the incorporation of data formerly stored only in departmental systems, the replacement of legacy systems, the strategy for integration or replacement of systems encountered during organizational mergers and acquisitions, as well as a long-term plan for replacement of new systems being acquired when they reach the end of their life or support for them is terminated. Other factors to consider for specific types of enterprise image data include the purpose for which the image was recorded (e.g., diagnostic, procedural, evidentiary) as well as any legal retention prior requirements, which may be specialty-specific. I.e., perhaps not all data needs to be migrated or made available in the replacement system with the same level of accessibility.

The importance of reliable, clean image metadata efficiently accessible in a standard format is well recognized. For radiology PACS, the migration-related benefits of both encoding images in DICOM [147] and providing access to them using the DICOM network protocol [148] are recognized. The challenges of migrating images encoded in obsolete or proprietary formats include constraints on the utility of converted data in the absence of sufficient metadata [149] and the loss of information in translation [150]. These lessons are equally applicable to enterprise imaging. The decision to use native format encoding rather than DICOM, and to store the metadata in a proprietary database, even if the latter is accessible using XDS or FHIR services, may create difficulties for future migration that need to be carefully considered.

#### Decision Making Strategy

Organizations planning an Enterprise Imaging strategy or embarking on integration of imaging specialties not currently storing to a centralized platform can benefit from pre-determining deployment based upon their workflow drivers and existing acquisition device and information system capabilities or options. Table 6 is an example of a matrix outlining specific decisions used to guide imaging strategy. While this example may not represent the drivers and system or device capabilities associated with the every organization, it provides a customizable overview. It is an example of a methodology for decision-making, not a recommendation.

**Table 6:** Example decision matrix

	Device supports DICOM network C-STORE or DICOMweb STOW-RS	Devices supports sending DICOM file using proprietary protocol	Device stores DICOM or standard consumer format file to local or network drive	Device stores proprietary file to local or network drive
Order required (either for billing or technical reasons)	Order-based DICOM	Order-based DICOM	Order-based DICOM conversion or XDS +/− CDA encapsulation	Order-based digitize documents or convert digital files, and plan to replace device
No order required	Encounter-based DICOM	Encounter-based DICOM	Encounter-based DICOM Conversion or XDS +/− CDA encapsulation	Encounter-based digitize documents or convert digital files, and plan to replace device

#### Editing Content

In many cases, enterprise images require some editing prior to finalizing the content for the medical record. For still images, this may include cropping the image, adjusting the brightness or contrast, applying some color correction or image processing (e.g., sharpen), or obscuring a region (e.g., facial features). For video, it may be required to edit the full-length video into one or more concise clips or apply some compression.

Within clinical areas, there may be a requirement to maintain some content for a period of time, while “publishing” a defined set of content for use within the EMR. For example, it may be required to maintain a full surgical video for use by surgeons, using a specialized application, while also providing selected clips to the EMR for inclusion in the patient’s medical record.

The workflow as to whether this editing is applied at the time of capture (e.g., medical photo on a mobile device) or afterward (e.g., post surgery) may vary and should be understood.

The final presentation of the images may be created at the time of acquisition, rather than retrospectively. Given that in many cases the photos or movies are documenting a procedure or status of the patient during the visit, it is important to save the “as viewed” images, with key images flagged.

If images are acquired raw, then there are the additional steps of post processing and creating the “for presentation” processed images that are then saved for ongoing general use. The original raw images may be saved as well but should be flagged as “for processing.” Note however that outside of DICOM there is no formal linkage between “for presentation” and “for processing” images. Having two versions of the same image can lead to confusion for later users and raises some patient safety/risk concerns regarding confusion of images, and time points or references.

In some cases such as surgery or endoscopic videos, there may be a long, constant recording during the entire procedure. Some systems provide the ability to edit out clips or still images but this is a time consuming process and can entail an additional loss in image quality due to decompressing a recompressing the images. Strong consideration should be given to a workflow that is to “capture only what you intend to keep.”

#### De-identification

For some use cases, such as teaching and research, information that may potentially identify the patient, or potentially be used in conjunction with other information to re-identify the patient, needs to be removed.

For most radiology and cardiology use cases, such information is usually confined to the DICOM metadata, though for some modalities it may be burned into the pixel data and need to be edited out, manually or automatically.

In some cases, the images themselves may be identifiable. It has been suggested that high resolution CT and MR images that include the face might be recognizable using manual or automated methods [151–153].

The incorporation of visible light images in enterprise systems increases the risk that identifying information might be present in the metadata (even of consumer formats like JPEG), as well as present in the pixel data (e.g., when labels are present in the photograph to identify the patient).

Full-face photographs and any comparable images are explicitly mentioned in the US HIPAA Privacy Rule [154]. Manual processes and photo editing tools are necessary to ensure de-identification of identifiable patient features within a medical photograph.

A special consideration for ophthalmology images is that retinal and iris images may also be inherently identifying, since they are used in biometric identification systems [155].

The normal considerations for metadata de-identification apply equally to enterprise imaging [156, 157]. Another special case unique to visible light images that may be overlooked is the presence of geolocation information (such as GPS coordinates) that may be present in the EXIF headers of JPEG files.

## Conclusion

At the beginning of this paper, the question was posed as to whether a pure DICOM solution is sufficient, or a hybrid approach is needed, which supports DICOM and non-DICOM payloads.

There is no clear industry consensus on this; different enterprises have made different choices in this regard and indeed the contributors to this paper cannot reach agreement on this matter. We are left to conclude that either approach is workable, since DICOM-based systems can encapsulate and regurgitate native formats and native formats can have their accompanying metadata stored and indexed in proprietary databases but accessed using standard protocols.

Comparable levels of functionality can be achieved with either DICOM or non-DICOM payloads, and other factors need to be considered carefully, including the capabilities of the installed base of systems with which the enterprise solution is being integrated, the current formats used in pre-existing archives and departmental systems being replaced, the strategies planned for exchange beyond the enterprise, as well as the current and future migration strategies being envisaged.

## Glossary

Application Programming Interface (API): A set of routines, protocols, and tools for building software applications [158].
Clinical Document Architecture (CDA): A document markup standard that specifies the structure and semantics of clinical documents for the purpose of exchange between healthcare providers and patients [159].
Cross-Enterprise Document Reliable Interchange (XDR): An IHE integration profile that provides document interchange using a reliable messaging system. This permits direct document interchange between EHRs, PHRs, and other healthcare IT systems in the absence of a document sharing infrastructure such as XDS Registry and Repositories [160].
Cross-Enterprise Document Reliable Interchange of Images (XDR-I): An IHE content profile for Imaging Document Object transmission using a point-to-point reliable messaging system specified Cross-Enterprise Document Reliable Interchange (XDR) [161].
Cross-Enterprise Document Sharing (XDS): An IHE integration profile that facilitates the registration, distribution, and access across health enterprises of patient electronic health records [162].
Cross-Enterprise Document Sharing for Imaging (XDS-I): An IHE integration profile that extends XDS to share images, diagnostic reports, and related information across a group of care sites [163].
Cross-Enterprise Sharing of Scanned Documents (XDS-SD): An IHE integration profile that associates structured, healthcare metadata with non-healthcare-specific document formats to maintain the integrity of the patient health record as managed by the source system [164].
CSIDQ: A complete set of images of diagnostic quality [19].
Diagnostically Acceptable Image Compression (DAIC): The use of lossy compression schemes and parameters for which it has been determined that for a particular type of image and a particular family of diagnostics tasks, compression does not adversely affect performance [114].
Digital Imaging and Communications in Medicine (DICOM®): The standard for the communication and management of medical imaging information and related data [165].
DICOMweb™: A term applied to the family of RESTful DICOM services defined for sending, retrieving, and querying for medical images and related information [166].
DNG: Adobe Digital Negative Format, a non-proprietary file format for storing camera raw files that can be used by a wide range of hardware and software vendors [167].
EHR: An Electronic Healthcare Record is an electronic version of a patient’s medical history that is maintained by the provider over time, and may include all of the key administrative clinical data relevant to that persons care under a particular provider, including demographics, progress notes, problems, medications, vital signs, past medical history, immunizations, laboratory data, and radiology reports [168].
EMPI: An Enterprise Master Patient Index is a database that is used across a healthcare organization to maintain consistent, accurate, and current demographic and essential medical data on the patients seen and managed within its various departments [169].
EMR: An Electronic Medical Record is a digital version of a paper chart that contains all of a patient’s medical history from one practice; contains the standard medical and clinical data gathered in one provider’s office [170].
Encounter-Based Workflow: Images are acquired during a clinic visit or procedure when image content acquisition is not considered the purpose of the visit [5].
Enterprise Image Repository (EIR): A standards-based DICOM and non-DICOM clinical image and video storage repository, with an index of the image and meta-information content, which is modality, modality vendor, specialty and service line, and viewer agnostic (adapted from [3]).
Enterprise Imaging: A set of strategies, initiatives, and workflows implemented across a healthcare enterprise to consistently and optimally capture, index, manage, store, distribute, view, exchange, and analyze all clinical imaging and multimedia content to enhance the electronic health record [3].
Enterprise Imaging Platform (EIP): A standards-based, enterprise infrastructure to support departmental imaging workflows, which includes modality work list services, image archival, index, enterprise viewer application viewing within or outside the EHR, query and retrieval of imaging content from most departments, as well as image exchange capabilities (adapted from [3]).
FHIR®: A set of Resources that represent granular clinical concepts. Designed for the web; the resources are based on simple XML or JSON structures, with an http-based RESTful protocol where each resource has predictable URL. Where possible, open internet standards are used for data representation [171].
Imaging Study: See Study.
Imaging Object Change Management (IOCM): An IHE integration profile that specifies how one actor communicates local changes applied on existing imaging objects to other actors that manage copies of the modified imaging objects in their own local systems. The supported changes include (1) object rejection due to quality or patient safety reasons, (2) correction of incorrect modality work list entry selection, and (3) expiration of objects due to data retention requirements. It defines how changes are captured and how to communicate these changes [146].
Import Reconciliation Workflow (IRWF): An IHE integration profile that manages importing images from CDs, hardcopy, etc. and reconciling identifiers to match local values [17].
Interoperability: The ability of two or more systems or components to exchange information and to use the information that has been exchanged [172].
Metadata: Data that describes other data [173]. In the context of imaging, it includes data that describes the pixel data (e.g., rows, columns) and data that describes the acquisition process (e.g., device, camera settings, date and time, location). In the context of medical imaging, it includes data that describes the patient (e.g., name, ID), workflow context (e.g., accession number), and clinical context (e.g., anatomy, functional conditions, observations, diagnosis).
Order-Based Workflow: Images are acquired as a result of an order placed in the managing information system [5].
Patient Demographics Query (PDQ): An IHE integration profile lets applications query a central patient information server and retrieve a patient’s demographic and visit information [105].
Patient Identifier Cross-Referencing (PIX): An IHE integration profile that supports the cross-referencing of patient identifiers from multiple Patient Identifier Domains by transmitting patient identity information from an identity source to the Patient Identifier Cross-reference Manager and providing the ability to access the list(s) of cross-referenced patient identifiers either via a query/response or via an update notification [174].
Radiology Information System (RIS): The core system for the electronic management of imaging departments. The major functions of the RIS can include patient scheduling, resource management, examination performance tracking, examination interpretation, results distribution, and procedure billing [175].
Study: A collection of one or more series of medical images, presentation states, and/or SR documents that are logically related for the purpose of diagnosing a patient. A study may include composite instances that are created by a single modality, multiple modalities or by multiple devices of the same Modality [176].
Vendor Neutral Archive (VNA): A medical imaging technology in which images and documents (and potentially any file of clinical relevance) are stored (archived) in a standard format with a standard interface, such that they can be accessed in a vendor-neutral manner by other systems [177].
Visible Light (VL) Imaging: Acquisition of images that are acquired by means of a camera or other sensors that are sensitive to visible or near-visible light [178].
